# hnRNPH2 as an Inhibitor of Chicken MDA5-Mediated Type I Interferon Response: Analysis Using Chicken MDA5–Host Interactome

**DOI:** 10.3389/fimmu.2020.541267

**Published:** 2020-10-06

**Authors:** Xian Lin, Shiman Yu, Haiying Mao, Peilei Ren, Meilin Jin

**Affiliations:** ^1^State Key Laboratory of Agricultural Microbiology, Huazhong Agricultural University, Wuhan, China; ^2^Department of Preventive Veterinary Medicine, College of Animal Medicine, Huazhong Agricultural University, Wuhan, China; ^3^Department of Biotechnology, College of Life Science and Technology, Huazhong Agricultural University, Wuhan, China; ^4^Key Laboratory of Development of Veterinary Diagnostic Products, Ministry of Agriculture, College of Veterinary Medicine, Huazhong Agricultural University, Wuhan, China; ^5^The Cooperative Innovation Center for Sustainable Pig Production, Wuhan, China

**Keywords:** chicken MDA5, interactome, chicken IFN-β, HNRNPH2, immune regulation

## Abstract

RIG-I and MDA5 are two key pattern recognition receptors that sense the invasion of RNA viruses and initiate type I interferon (IFN) response. Although these receptors are generally conserved in vertebrates, RIG-I is absent in chickens, whereas MDA5 is present. Chicken MDA5 (chMDA5) plays a pivotal role in sensing the invasion of RNA viruses into cells. However, unlike mammalian MDA5, where there are in-depth and extensive studies, regulation of the chMDA5-mediated signaling pathway remains unexplored. In this study, we performed a pulldown assay and mass spectrometry analysis to identify chicken proteins that could interact with the N terminal of chMDA5 (chMDA5-N) that contained two CARDs responsible for binding of the well-known downstream adaptor MAVS. We found that 337 host proteins could potentially interact with chMDA5-N, which were integrated to build a chMDA5-N–host association network and analyzed by KEGG pathway and Gene Ontology annotation. Results of our analysis revealed that diverse cellular processes, such as RNA binding and transport and protein translation, ribosome, chaperones, and proteasomes are critical cellular factors regulating the chMDA5-mediated signaling pathway. We cloned 64 chicken genes to investigate their effects on chMDA5-mediated chicken IFN-β production and confirmed the association of chicken DDX5, HSPA8, HSP79, IFIT5, PRDX1, and hnRNPH2 with chMDA5-N. In particular, we found that chicken hnRNPH2 impairs the association between chMDA5-N and MAVS and thus acts as a check on the chMDA5-mediated signaling pathway. To our knowledge, this study is the first to analyze the chicken MDA5–host interactome, which provides fundamental but significant insights to further explore the mechanism of chicken MDA5 signaling regulation in detail.

## Introduction

In higher vertebrates, genome-encoded recognition receptors sense the non-self component of invading viruses and initiate the antiviral innate immune response. The response is characterized by the induction of type I interferon (IFN) and pro-inflammatory cytokines, which resists virus replication (1). Retinoic acid-inducible gene I (RIG-I)-like receptors (RLRs), such as RIG-I (DDX58), MDA5 (IFIH1), and LGP2 (DHX58), can detect RNA viruses as non-self patterns in the cytoplasm of most cell types. These RLRs have a DExD/H-box RNA helicase domain and a C-terminal domain (CTD) that is responsible for binding viral RNA (2). In addition, RIG-I and MDA5 harbor two N-terminal caspase activation and recruitment domains (CARDs), which are important for interaction with the downstream adaptor molecule, MAVS/IPS-1 (3).

RIG-I and MDA5 differ in terms of recognizing viral RNA species (4): RIG-I mainly senses RNA with a panhandle structure and a 5′ triphosphate moiety, whereas MDA5 senses long dsRNA or web-like RNA aggregates (5–7). RIG-I detects orthomyxoviruses, rhabdoviruses, and arenaviruses, whereas MDA5 detects positive-strand RNA viruses, particularly *Picornaviridae* and *Flaviviridae* members (8–10). Upon recognition of RNA viruses, RIG-I and MDA5 bind to their adaptor molecule MAVS/IPS-1 through CARD–CARD association, an essential step for the activation of downstream signaling. When RIG-I and MDA5 interact with MAVS, several well-studied kinases of the IKK family, namely, IKKε, TBK1, and IKKα/β/γ, are activated, which phosphorylates transcription factors IFN regulatory factor 3 and 7 (IRF3/7), eventually inducing IFN transcription. Studies on mammalian cells revealed that activation of RIG-I and MDA5 takes place in a multistep process consisting of viral RNA binding, conformational changes, and a series of posttranslational modifications (PTMs). RIG-I is autorepressed in resting cells because of the masking of CARDs by the CTD that prevents the CARD–CARD association between RIG-I and MAVS (11). Although the CTD exists also in MDA5, it does not exhibit any repressor function. It seems that in the absence of RNA viruses, MDA5 activity is controlled by other mechanisms. RIG-I and MDA5 undergo phosphorylation at multiple residues in uninfected cells to prevent aberrant downstream signaling: S8 and T170 in the RIG-I CARD and S88 in the MDA5 CARD (12–14). In response to the binding of viral RNA, two phosphoproteins (PP1α and PP1γ) dephosphorylate RIG-I (S8 and T170) and MDA5 (S88) in the CARDs, thereby allowing the MAVS CARDs to bind with them and trigger downstream signaling (14). In RIG-I, dephosphorylation triggers ubiquitination of the robust K63 by two critical ubiquitin E3 ligases, TRIM25 (15) and Riplet (16), and ultimately leads to RIG-I oligomerization and MAVS binding. However, the role of K63-linked ubiquitin polymers in MDA5 activation is still under debate. Many host proteins, such as Sec14L1 (17), ATG5–ATG12 (18), and NLRX1 (19), can regulate RIG-I signal transduction and thus disrupt the association between RIG-I and MAVS. These proteins that disrupt the CARD–CARD association can also hinder RIG-I and MDA5 signal transduction.

Although RLRs are generally conserved in vertebrates, the RIG-I gene is absent in chickens (20). However, chicken cells have an intact MDA5 gene; as a mammalian MDA5, chicken MDA5 (chMDA5) can also mediate type I IFN response (21–23). The N-terminal 1–483 amino acid of chMDA5 (chMDA5-N) contains two CARDs and can be exploited as an efficacious adjuvant for a vaccine against the lethal H5N1 influenza virus by inducing IFN-β production (24). chMDA5-N can also enhance the immune response to inactivated Newcastle disease virus (NDV) (25). Chicken MAVS is essential to the chMDA5-mediated type I IFN induction downstream of chMDA5 and upstream of chicken IRF7 (21). This observation indicates that the pathway downstream of chMDA5 is intact in chicken cells. Thus, chMDA5 could play a pivotal role in sensing RNA virus infection in chickens. However, the regulation of chMDA5 activation has not been studied yet, thus restricting our understanding of the mechanisms of chicken innate antiviral response and the role of chMDA5 signaling in combating chicken RNA viruses. In the present work, we aimed at identifying the protein association network between chMDA5 and chicken cells and elucidating the cellular factors that regulate chMDA5 signaling using mass spectrometry. We found that diverse cellular processes, such as RNA binding and ubiquitination, chaperones, and RNA translation, are involved in regulating the chMDA5-mediated signaling pathway. In particular, chicken hnRNPH2 is identified as a negative regulator of chMDA5 signaling as it disrupts the association between chMDA5 and chicken MAVS. To our knowledge, this research is the first report on the chMDA5–host interactome and will provide a foundation for further investigations into chMDA5 signaling regulation.

## Materials and Methods

### Cells and Virus

Chicken cell line DF1 was propagated in Dulbecco’s minimal essential medium (Hyclone) supplemented with 10% heat-inactivated fetal bovine serum (Hyclone) and incubated at 39°C in a humidified incubator with 5% CO_2_. A highly pathogenic avian influenza virus A/duck/Hubei/WH18/2015 (H5N6) was conserved in our laboratory and propagated in allantoic cavities of 9- to 11-day-old fertile SPF chicken eggs. Experiments with the H5N6 virus were conducted in an Animal Biosafety Level 3 laboratory, Huazhong Agricultural University, China, in compliance with the institutional biosafety manual.

### Antibodies, RNA Synthesis, and Transfection Reagent

The following antibodies were used for immunoblotting: anti-Flag (M2; 1:10,000; Sigma), anti-HA (1:5000; Abclone), anti-GST (1:2000; Abclone), anti-hnRNPH2 (1:1000; Abclone), anti-GAPDH (1:5000; Proteintech), anti-PB2 and anti-NP (1:5,000; GeneTex), anti-mouse/rabbit IgG, HRP-linked secondary antibodies (Proteintech), anti-mouse IgG (Alexa Fluor 488 conjugate; Cell Signaling), and anti-rabbit IgG (Alexa Fluor 594 conjugate; Proteintech). A specific siRNA targeting the chicken hnRNPH2 sequence (5′ CCGCGGATATAACAGTCTT 3′) was synthesized using RiboBio (Guangzhou, China). Lipo8000 (Byotime Biotechnology) was used to transfect plasmids and RNA according to the manufacturer’s instructions. Lipo8000 in a final concentration of 80 nM per well was used for transfection of siRNA specific for hnRNPH2. Non-targeting siRNA control (NC) (RiboBio) was used as control.

### Gene Cloning and Plasmid Construction

All genes were cloned from cDNA obtained from DF1 cells into eukaryotic expression vectors such as pCMV14-3Flag, pCAGGS-HA, or pCDNA3.1-GST.

### GST Pulldown Assay and Mass Spectrometry

DF1 cells were taken in ten 15-cm dishes and were transfected with 15 μg/dish empty vector pDNA3.1-GST or pCDNA3.1-GST–chMDA5-N (total, 150 μg) to purify chMDA5-N complexes using the GST pulldown technique. The cells were harvested 24 h posttransfection, washed in ice-cold phosphate buffer saline (PBS), and pelleted at 450 g for 5 min at 4°C. They were then lysed on a rotating wheel for 30 min at 4°C in RIPA buffer (Sigma) with ETDA-free protease inhibitor cocktail and phosphatase inhibitor (Sigma). The soluble fraction was separated by centrifugation at 13,000 × *g* for 10 min at 4°C. Cleared lysates were mixed with glutathione–agarose beads (Pierce) according to the manufacturer’s instructions. After being extensively washed with washing buffer, the proteins bound to the glutathione beads were eluted using a buffer containing reduced glutathione (Thermo Fisher Scientific). A 10% (vol/vol) volume of the eluted proteins was separated on 10% SDS–PAGE gel. After silver staining, the protein bands in the GST–chMDA5-N group different from those in control were excised and analyzed using liquid chromatography–tandem mass spectrometry (LC–MS/MS) at PTM Bio Company (Hangzhou, China). Another 10% (vol/vol) volume of the eluted proteins was subjected to LC–MS/MS to identify co-immunoprecipitated host proteins. The remaining 80% (vol/vol) of the eluate was analyzed using LC–MS/MS without separation. The MS/MS signals were processed against the Uniprot Gallus protein database (36624) using the Mascot algorithm with the following parameters: variable modifications; oxidation (Met); N-acetylation; pyroglutamination (Gln); maximum missed cleavages, 2; peptide mass tolerance, 100 ppm; and MS/MS tolerance, 0.5 Da. Protein identification was based on the criterion of having at least one MS/MS data signal with Mascot scores that exceeded the threshold (*p* < 0.05).

### Analysis of Association Proteins

After the proteins identified in the control group were removed, the remaining proteins in the elution of GST–chMDA5-N were labeled chMDA5-N association proteins. In the differential bands separated by SDS–PAGE, all the identified proteins were considered to be chMDA5-N association proteins. Both of these proteins were merged and analyzed to construct an association network using STRING^1^. Protein–protein associations were then illustrated using Cytoscape, version 3.7.1. Gene Ontology (GO) and KEGG were also analyzed using STRING.

### Co-immunoprecipitation and Western Blotting

DF1 cells were transfected with the indicated tagged vectors using the Lipo8000 transfection reagent. The cells were then incubated in RIPA buffer with EDTA-free protease inhibitor cocktail for 30 min at 4°C 24 h later. After centrifugation to remove cellular debris, the supernatant was incubated overnight with an anti-Flag- or anti-HA-tagged magnetic beads (Bimake) for 6 h at 4°C. The beads were washed thrice with cold lysis buffer and twice with cold PBS. The proteins bound to the beads were mixed with loading buffer (Sigma), boiled for 10 min, and then subjected to SDS–PAGE, followed by Western blotting. For Western blotting, the proteins separated by SDS–PAGE were transferred electrophoretically to a nitrocellulose filter membrane (NC) membrane (GE Technology) in transfer buffer [100 mM Tris, 190 mM glycine, 10% (vol/vol) methanol]. After blocking for 1 h, the membrane was incubated with the indicated primary antibodies for 2 h at room temperature, followed by three washes with PBS plus Tween 20 (PBST). As the final step, the membrane was reacted with the indicated secondary antibodies for 1 h at room temperature and washed thrice with PBST for 15 min. Specific signals were visualized using the Western blot ECL reagent (Advansta).

### Dual-Luciferase Reporter Assay

DF1 cells were transfected in 12-well plates with 0.5 μg of pCAGGS–chMDA5-N or pCAGGS–chMDA5. The amount of 0.5 μg indicated chicken gene expression plasmids together with 0.5 μg of chicken IFN-β (chIFN-β) promoter reporter plasmids chIFN-β-luc and 10 ng of internal control Renilla (PGL4.75 hRluc/CMV; Promega) were transfected into DF1 cells in 12-well plate. After transfection for 24 h, the cells were lysed, and firefly and Renilla luciferase activities were measured in accordance with the manufacturer’s instructions. All the obtained luciferase values were normalized against those of the Renilla luciferase control. For each experiment, at least three independent measurements were performed, and each experiment was performed in triplicate.

### Quantitative Real-Time PCR Assay

Total RNA was extracted using TRIzol, and 1 μg of RNA was reverse-transcribed using All-in-One cDNA Synthesis SuperMix (Bimake). The relative mRNA expression was calculated by SYBR Green-based quantitative real-time PCR (qRT-PCR) technique using SYBR Green SuperMix (Bimake) in an ABI ViiA 7 PCR System (Applied Biosystem) using *Actin-*β as the reference gene, in relation to the control samples. Specific primers for qRT-PCR are listed in Table 1.

**TABLE 1:** Primers for qRT-PCR.

Primers	Sequences (5′→3′)
chIFN-β-F	AGCTCTCACCACCACCTTCTC
chIFN-β-R	TGGCTGCTTGCTTCTTGTCCTT
chHNRNPH2-F	TCACTACGACCCTCCACGCAAG
chHNRNPH2-R	GCCTCATCCTCTCCAAGCCACT
NP-F	AGCAATGATGGATCAAGTGCGAGAG
NP-R	AAGCAGGCAAGCAGGACTTATGG
chActin-β-F	AAATTGTGCGTGACATCAAGGA
chActin-β-R	AGGCAGCTGTGGCCATCTC

### Statistical Analysis

Results are expressed as mean ± standard deviation (SD), and all data are representative of no less than three independent experiments. The data were analyzed using unpaired Student’s *t*-test; *p* < 0.05 was considered significant.

## Results

### Chicken MDA5 (chMDA5)–Host Interactome Analysis

**FIGURE 1:**
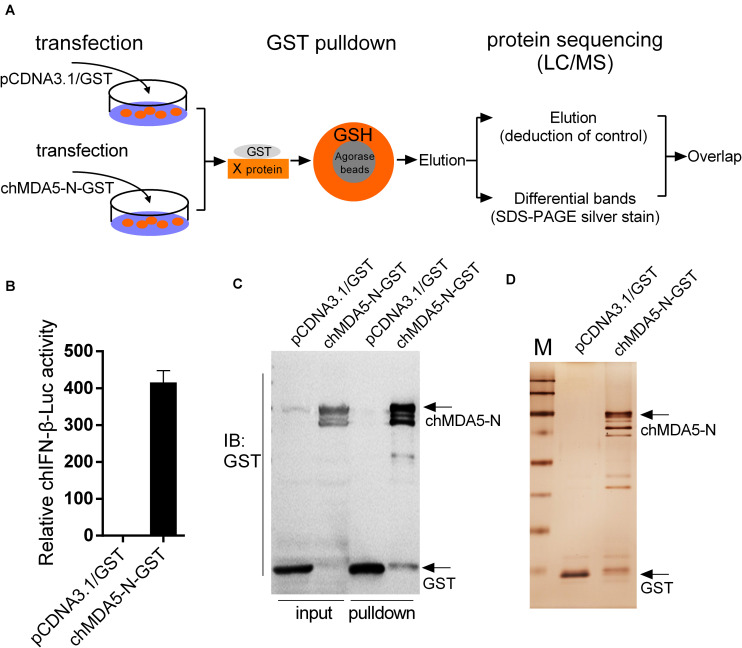
Isolation of chMDA5-N and the associated host proteins from DF1 cells. **(A)** DF1 cells were transfected with pCDNA3.1/GST (empty vector) or pCDNA3.1/GST–chMDA5-N.chMDA5-N complexes were purified using the GST pulldown technique 24 h later, followed by mass spectrometry analysis as depicted in the figure. **(B)** GST–chMDA5-N or control vector was transfected into DF1 cells, together with chicken IFN-β (chIFN-β)-luc and internal control Renilla. The cells were lysed 24 h later for firefly and Renilla luciferase activity determination. The proteins obtained from the GST pulldown assay in **(A)** were separated using SDS–PAGE followed by Western blotting to detect GST and GST–chMDA5-N **(C)** and silver staining **(D)** to evaluate the quality of the GST pulldown. The data in **(B)** are means and standard deviations (SD) of three biological repeats.

GST pulldown assay and subsequent mass spectrometry were conducted to identify chMDA5-interacted host proteins. chMDA5 possesses two CARDs in the N terminal, which is essential for downstream signal activation. Thus, we constructed GST–chMDA5-N containing two CARDs to pull down its association proteins by transfecting it with DF1 cells, as shown in Figure 1A. GST–chMDA5-N could significantly activate chIFN-β transcription according to promoter reporter assay (Figure 1B). We then evaluated the quality of the GST pulldown by detecting GST–chMDA5-N and the proteins that interacted with it using Western blot (Figure 1C) and silver staining (Figure 1D), respectively, and then identified the proteins in elution and differential bands using mass spectrometry (Figure 1A). This showed that chMDA5-N was significantly enriched after the pulldown; besides, many visible bands possibly interacting with chMDA5-N were observed in the chMDA5-N GST pulldown group. The data were then analyzed as described in the section “Materials and Methods.” A total of 201 and 207 proteins were identified in the elution (excluding control) and differential band groups, respectively, with 70 proteins overlapping in the two groups (Supplementary Table S1). A total of 337 proteins were identified using the mass spectrometry analysis of the samples.

**FIGURE 2:**
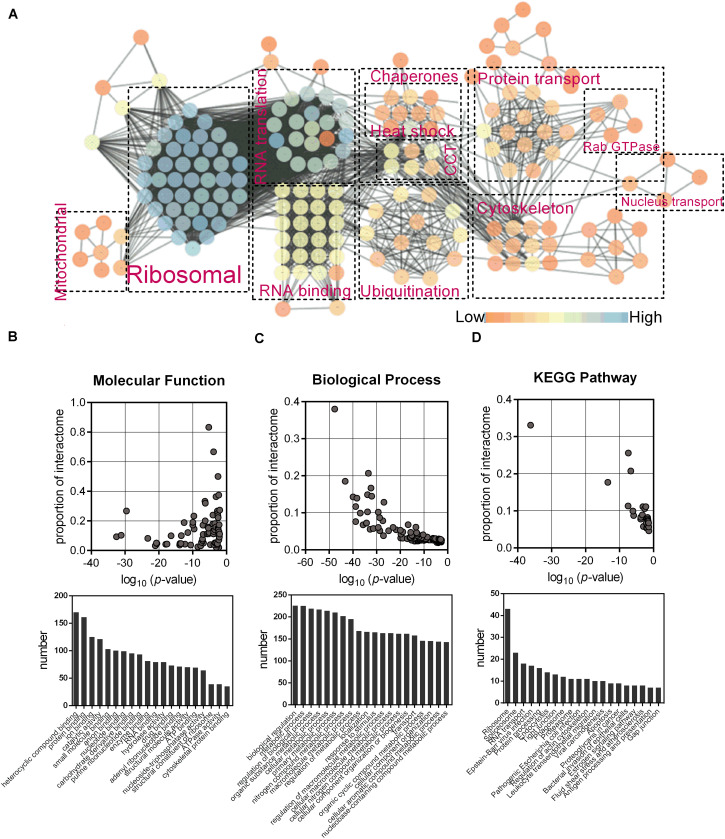
Identification of the interactome of chMDA5-N. **(A)** Association network of host proteins that were potentially associated with chMDA5-N, which was based on STRING database and illustrated using Cytoscape, version 3.7.1. Abundant proteins were manually sorted into the indicated categories. Different colors of nodes indicated their connectivity in the network, as shown in the figure. Gene Ontology molecular function terms **(B)**, and Biological process terms **(C)**, and KEGG pathway **(D)** were also analyzed using STRING. The probability that the terms were enriched in the interactome was calculated by comparing them with the annotation of the total chicken proteome. Only the top 20 terms based on the numbers assigned to each term are listed.

The association network was constructed using STRING and Cytoscape (Figure 2A), which showed nine enriched categories, namely, ribosomal, RNA translation, RNA binding, chaperones, ubiquitination, protein transport, cytoskeleton, nucleus transport, and mitochondrial. Most of the bound proteins could interact with at least one of the other bound proteins, suggesting that complexes of interesting proteins may have been purified. GO mapping showed that the interactome was highly enriched with proteins of various molecular functions and biological processes (Figures 2B,C). KEGG analysis indicated that ribosome, RNA transport, protein processing, spliceosome, proteasome, and cytoskeleton were significantly enriched (Figure 2D).

### Influence of Cloned Chicken Genes on Chicken IFN-β (chIFN-β) Promoter Activity

**FIGURE 3:**
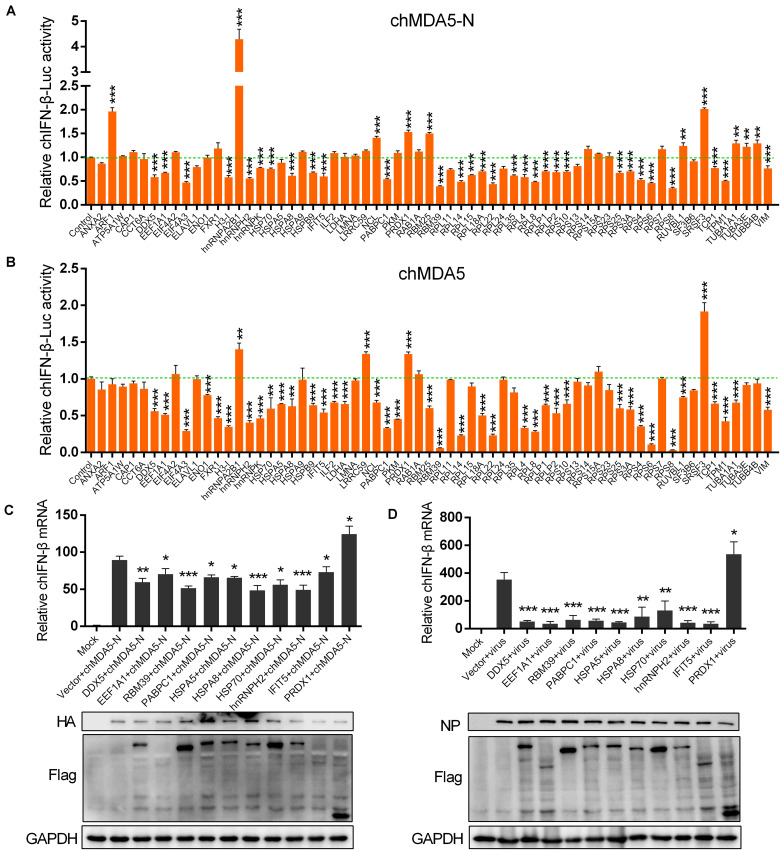
Influence of chicken proteins selected from the interactome on chicken IFN-β (chIFN-β) production. DF1 cells in 12-well plates were co-transfected with 0.5 μg of pCAGGS–chMDA5-N **(A)** or pCAGGS–chMDA5 **(B)** and 0.5 μg of genes that were cloned into expression vector pCMV14-3Flag, together with 0.2 μg of chIFN-luc and 10 ng internal control Renilla. The cells were lysed for firefly and Renilla luciferase activities 24 h later. DF1 cells in a 12-well plate were transfected with 0.5 μg of pCMV14-3Flag vector expressing the indicated chicken genes, together with 0.5 μg of pCAGGS–chMDA5-N. Total RNA was extracted for chIFN-β mRNA determination 24 h later using quantitative real-time PCR (qRT-PCR) **(C)**. DF1 cells in a 12-well plate were transfected with 1 μg of pCMV14-3Flag vector expressing the indicated chicken genes. The cells were infected with H5N6 virus 24 h later (MOI = 1.0); 16 h later, total RNA was extracted for chIFN-β mRNA determination using qRT-PCR **(D)**. The transfected gene or viral replication was confirmed by western blotting using corresponding antibodies. Data shown are means and SD, and one representative of three independent experiments. *p < 0.05, **p < 0.01, ***p < 0.001 (unpaired *t*-test).

We constructed 64 chicken gene expression plasmids from the proteins identified by the mass spectrometry analysis to investigate their potential regulation on chIFN-β production using luciferase reporter assay. According to the GO categories, we selected genes associated with ribosome such as RPS and RPL; RNA binding such as hnRNPH2, hnRNPA2B1, and RBM39; RNA translation such as EEF1A1 and EIF4A2; spliceosome such as SRSF3 and SF3B6; chaperone such as HSP70 and HSPA8; ubiquitination such as TUBA1A1 and TUBA3E; and some other proteins such as PRDX1, DDX5, and IFIT5. We co-transfected the chicken genes with chMDA5-N or chMDA5 to evaluate the influence of the cloned genes on the chIFN-β promoter activity. All the transfected genes were successfully expressed in DF1 cells (Supplementary Figure S1). Results indicated that 39 genes could significantly regulate the chMDA5-N-mediated chIFN-β promoter activity, with 10 genes (i.e., ARF1, hnRNPA2B1, NCL, PRDX1, RBM25, SRSF3, RUVBL3, TUBA1A1, TUBA3E, and TUBB4B) enhancing the chIFN-β promoter activity (Figure 3A); 42 genes could significantly regulate the chMDA5 (full-length)-mediated chIFN-β promoter activity (Figure 3B), with four genes (i.e., hnRNPA2B1, LRRC59, PRDX1, and SRSF3) enhancing the chIFN-β promoter activity. A total of 31 genes could regulate both chMDA5-N-mediated and chMDA5 (full-length)-mediated chIFN-β promoter activity in a similar way. However, some of the genes could regulate the chMDA5-N-mediated chIFN-β promoter activity, but not the chMDA5 (full-length)-mediated one, such as ARF1 TUBA1A1, TUBA3E, and TUBB4; some of the genes that influenced the chMDA5 (full-length)-mediated chIFN-β promoter activity did not regulate the chMDA5-N-mediated chIFN-β promoter activity, such as ENO1, FXR1, HSPA5, and PKM. This difference could be due to that chMDA5 (full-length) contained not only the CARD on the N terminal, but also the CTD (C-terminal domain), which could be influenced by specific chicken proteins. We then investigated the regulation of the 10 genes that could significantly regulate the chIFN-β promoter activity mediated by both chMDA5-N and chMDA5 transfection on the chIFN-β mRNA level induced by chMDA5-N transfection (Figure 3C) or by avian influenza virus H5N6 infection (Figure 3D) in DF1 cells using qRT-PCR. Results showed that overexpression of nine genes (i.e., DDX5, EEF1A1, RBM39, PABPC1, HSPA5, HSPA8, HSP70, hnRNPH2, and IFIT5) could remarkably decrease, whereas PRDX1 could significantly increase chIFN-β expression, stimulated by chMDA5-N transfection and H5N6 virus infection. This indicated that these 10 genes play an important role in the regulation of chMDA5-N-mediated chIFN-β production.

### Association of Six Host Proteins With chMDA5-N

**FIGURE 4:**
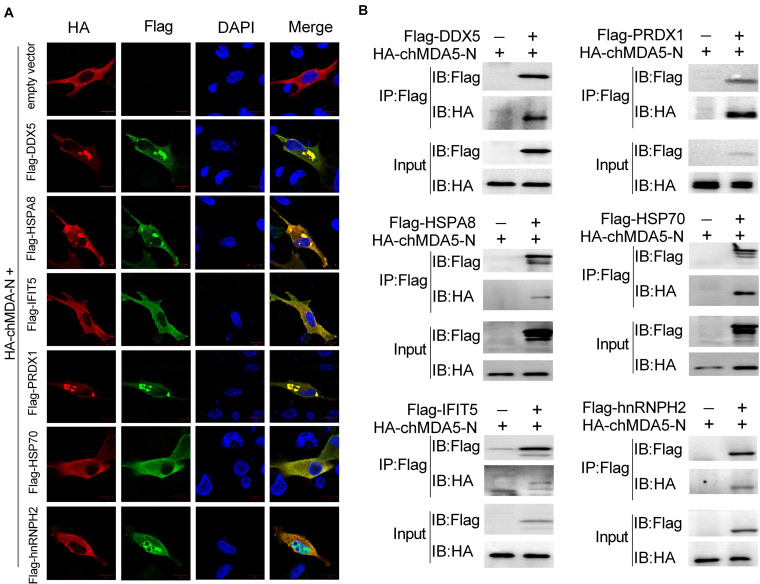
Validation of association between chMDA5-N and chicken proteins. **(A)** Confocal microscopy analysis of co-localization of chMDA5-N and six indicated chicken proteins. DF1 cells were transfected with pCAGGS–chMDA5-N and the indicated chicken proteins with a Flag tag for 24 h, and then immunostaining of HA–chMDA5 (red) and Flag (green) was performed. **(B)** 1 μg of HA-tagged chMDA5-N and 1 μg of Flag-tagged indicated chicken proteins were co-transfected into DF1 cells grown in a six-well plate. Total proteins were extracted 24 h later using RIPA buffer, followed by co-immunoprecipitation (Co-IP) and immunoblotting analysis.

We speculated that the proteins that significantly regulated chIFN-β as shown in Figure 3 would be associated with chMDA5-N. We examined the 10 proteins as shown in Figure 3C and found that six (i.e., DDX5, HSPA8, IFIT5, PRDX1, HSP70, and hnRNPH2) of them could co-localize well with chMDA5 when co-transfected into DF1 cells (Figure 4A). DDX5, HSPA8, IFIT5, PRDX1, and HSP70 were mainly distributed across the cytoplasm, whereas hnRNPH2 was distributed in both the nucleus and the cytoplasm. All the co-localization occurred in the cytoplasm, in accordance with the location of chMDA5, which functions mainly in the cytoplasm. In particular, DDX5, HSPA8, and PRDX1 formed clear aggregations with chMDA5-N in the cytoplasm. We performed co-immunoprecipitation (Co-IP) assays to further confirm their associations, which showed that chMDA5-N could obviously interact with DDX5, HSPA8, IFIT5, PRDX1, HSP70, and hnRNPH2 (Figure 4B). We performed additional co-IP experiments in DF1 cells. Cell lysates were immunoprecipitated with rabbit pAb against chMDA5, followed by western blotting with anti-DDX5, HSP70, HSPA8, hnRNPH2, IFIT5 and PRDX1 antibodies. As shown in Supplementary Figure S2, all of the six selected chicken proteins were able to be immunoprecipitated with chMDA5. These results suggested that these six proteins may play important roles in the chMDA5-mediated innate immune response by interacting with chMDA5-N, which deserves further investigation.

### Chicken hnRNPH2 Restricted chIFN-β Production by Targeting chMDA5

**FIGURE 5:**
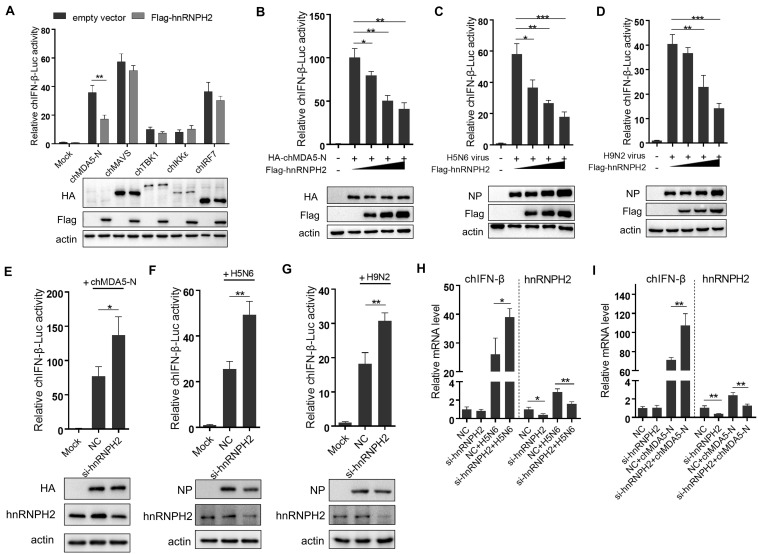
Chicken hnRNPH2 inhibited chIFN-β production by targeting chMDA5. **(A)** Chicken IFN-β promoter activity was tested as described earlier. **(B)** 0.5 μg of HA-tagged chMDA5-N, 0.2 μg of chIFN-luc, 10 ng Renilla, and increasing amounts of chicken hnRNPH2 (i.e., 0, 0.2, 0.4, and 0.8 μg) were co-transfected into DF1 cells in a 12-well plate for 24 h, and then, the cells were lysed for luciferase test. **(C)** Increasing amounts of Flag-tagged chicken hnRNPH2 (i.e., 0, 0.2, 0.4, and 0.8 μg) were transfected into DF1 cells in a 12-well plate for 20 h and then left uninfected or infected with H5N6 (MOI = 2) **(C)** or H9N2 (MOI = 5) **(D)** for 12 h until luciferase assays. **(E)** 1 μg of HA-tagged chMDA5-N, 0.2 μg chIFN-luc, and 10 ng Renilla were co-transfected into DF1 cells in a 12-well plate for 8 h, which were then transfected with 80 nM siRNA targeting chicken hnRNPH2 for another 30 h until luciferase assays. DF1 cells in a 12-well plate were transfected with 80 nm siRNA targeting chicken hnRNPH2 for 24 h and then were infected with H5N6 (MOI = 2) **(F)** or H9N2 (MOI = 5) **(G)** for 12 h, followed by luciferase assays. **(H)** The cells were transfected with 1 μg of chMDA5-N for 8 h and then were transfected with 80 nM siRNA targeting chicken hnRNPH2 for another 30 h. Total RNA was then extracted for chIFN-β and chicken hnRNPH2 by mRNA analysis using qRT-PCR. **(I)** The cells were transfected with 80 nM siRNA targeting chicken hnRNPH2 for 24 h and then were infected with H5N6 (MOI = 2) for 12 h; then, chIFN-β and chicken hnRNPH2 mRNA analysis was performed using qRT-PCR. Data shown are means and SD, and one representative of three independent experiments. *p < 0.05, **p < 0.01, ***p < 0.001 (unpaired *t*-test).

Among the six proteins that could interact with chMDA5-N as shown in Figure 4, hnRNPH2 particularly gained our interest, which belongs to the hnRNP family of proteins with RNA binding function, because numerous studies have reported that other hnRNP family proteins could regulate immune response in mammalian cells, such as hnRNPA2B1 (26), hnRNPA1 (27), and hnRNPM (28). We investigated the influence of hnRNPH2 on five well-known key regulators (i.e., chMAVS, chTBK1, chIKKε, chIRF7, and chMDA5-N) that induced chIFN-β production, using chIFN-β promoter luciferase reporter assay. Expression of all five genes was found to significantly activate the chIFN-β promoter. However, hnRNPH2 was found to significantly inhibit the chMDA5-N-induced chIFN-β promoter activity (Figure 5A). It is likely that hnRNPH2 negatively regulates chIFN-β production mainly by targeting chMDA5. Further studies showed that hnRNPH2 could inhibit the chIFN-β promoter activity induced by chMDA5-N transfection (Figure 5B), H5N6, and H9N2 virus infection (Figures 5C,D) in a dose-dependent manner. On the contrary, knockdown of chicken hnRNPH2 by specific siRNA transfection significantly enhanced the chIFN-β promoter activity induced by chMDA5-N, H5N6 and H9N2 virus infection (Figures 5E–G). The increase in chIFN-β expression by the hnRNPH2 knockdown was further validated using qRT-PCR (Figures 5H,I). These findings clearly demonstrated that chicken hnRNPH2 could repress chIFN-β production by targeting chMDA5.

### Chicken hnRNPH2 Disrupted chMDA5–MAVS Association

**FIGURE 6:**
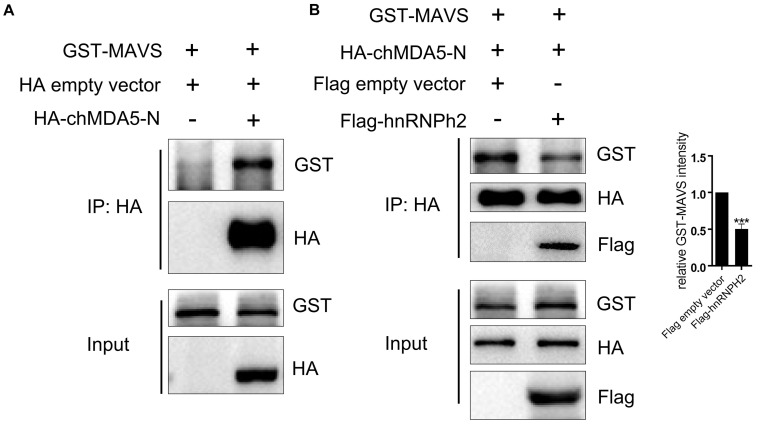
Chicken hnRNPH2 weakened the association between chMDA5-N and MAVS. **(A)** 1 μg of GST-tagged chicken MAVS was transfected into DF1 cells in a six-well plate together with 1 μg of HA-tagged chMDA5-N plasmid or empty vector. Total proteins were extracted using RIPA buffer 24 h later, followed by Co-IP and immunoblotting analysis. **(B)** 1 μg of GST-tagged chicken MAVS and 1 μg of HA-tagged chMDA5-N were co-transfected into DF1 cells in a six-well plate, together with 1 μg of Flag-tagged chicken hnRNPH2 or empty vector. Total proteins were extracted using RIPA buffer 24 h later, followed by Co-IP and immunoblotting analysis. The densitometry of GST–MAVS expression was quantified using ImageJ software, and control was set 1.00. Data are shown as one representative of three independent experiments. ***p < 0.001 (unpaired *t*-test).

MDA5 activates the downstream signaling pathway by interacting with the mitochondrial adaptor MAVS through CARD–CARD association. In this study, chicken hnRNPH2 was shown to interact with chMDA5-N, which contained two CARDs; this interaction seems to be independent of RNA binding of hnRNPH2, because Flag-hnRNPH2 was still co-immunoprecipitated with HA-chMDA5-N upon pretreatment of cell lysates with RNase A/T1 (Supplementary Figure S3); therefore, we supposed that hnRNPH2 would influence the association between chMDA5 and chicken MAVS. We first investigated the association of chMDA5-N with MAVS by co-transfecting them into DF1 cells using Co-IP and found that chMDA5-N interacted with chicken MAVS (Figure 6A). However, when chMDA5-N with a HA tag and MAVS with a GST tag were co-transfected into DF1 cells, together with hnRNPH2 with a Flag tag, the association between chMDA5-N and MAVS was obviously weakened compared with the empty vector transfection, as shown in Figure 6B. This result indicated that chicken hnRNPH2 could disrupt the chMDA5–MAVS association and thus repress the chIFN-β production.

### Chicken hnRNPH2 Promoted Avian Influenza Virus Replication

**FIGURE 7:**
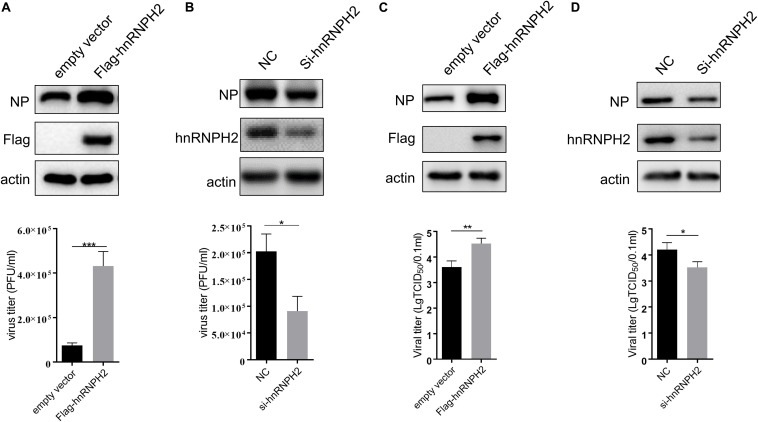
Chicken hnRNPH2 was beneficial for H5N6 virus replication. 1 μg of empty vector or Flag-tagged chicken hnRNPH2 **(A,B)** and 80 nm siRNA NC or siRNA specific for chicken hnRNPH2 **(C,D)** were transfected into DF1 cells in a 12-well plate for 24 h. The cells were then infected with H5N6 virus (MOI = 1) for 30 h. Total proteins were harvested for the indicated protein determination using Western blotting **(A,C)**, and the virus titer in the supernatant was determined using plaque assay. Data are shown as one representative of three independent experiments. *p < 0.05, **p < 0.01, ***p < 0.001 (unpaired *t*-test).

Given that chicken hnRNPH2 inhibits chMDA5-mediated signaling, we examined the roles of hnRNPH2 in cellular antiviral response. Replication of avian influenza virus H5N6 was markedly increased in chicken hnRNPH2-overexpressed DF1 cells compared with the control cells, as monitored by influenza viral protein PB2 and NP detection (Figure 7A). Plaque assays showed that viral titers of H5N6 were higher in hnRNPH2-overexpressed DF1 cells compared with empty-vector-transfected cells (Figure 7B). On the contrary, knockdown of chicken hnRNPH2 by specific siRNA transfection significantly suppressed the viral PB2 and NP protein expression (Figure 7C) and virus titers (Figure 7D). These results showed that chicken hnRNPH2 is a positive factor in avian influenza virus replication.

## Discussion

Mammals and birds diverged into different lineages approximately 300 million years ago. Although many evolutionarily conserved regions are present in their DNA, the avian genome contains many genes that are different from those of mammals (29). Avian and mammalian immune systems vary in many ways (30–32), such as the lack of eosinophils in the avian immune system and the avian-specific bursa of Fabricius that cannot be found in mammals. Although highly conserved RLRs are present in vertebrates, the substantial difference also exists between chickens and mammals. Chickens lack RIG-I but possess intact MDA5 and encode IRF7 instead of IRF3 to perform a similar function (20, 23, 33). Owing to the lack of RIG-I in chickens, chMDA5 plays a pivotal role in sensing RNA virus infection to initiate innate antiviral response. However, the regulation mechanism of chicken MDA5-mediated signaling pathway remains unexplored so far.

In contrast to the in-depth and extensive research of the RLR signaling pathway in mammalian cells, fundamental studies in chicken cells are not sufficient. With PTM as an example, mammalian MDA5 undergoes phosphorylation at S88 in CARDs to prevent aberrant downstream signaling. This phosphorylation is then inhibited by phosphatase 1 (PP1α and PP1γ) in response to viral RNA binding, thus allowing MAVS binding (14). However, whether this process occurs in chMDA5 is still unclear. In the present study, we revealed that the CARDs of chMDA5 could significantly activate chIFN-β production. We performed pulldown assay and mass spectrometry to identify factors that regulate chMDA5-mediated chIFN-β signaling. This experiment allowed us to construct a chMDA5–host interactome and provided several protein candidates for studying the mechanism of chMDA5-activated signaling pathway.

We found that the proteins associated with ribosome, RNA binding, protein translation, chaperones, ubiquitination, protein transport, cytoskeleton, nucleus transport, and mitochondria were significantly enriched. By examining the 64 chicken proteins identified using LC/MS, we found that many proteins could significantly regulate the chMDA5-mediated chIFN-β production. However, only a few proteins could enhance the chIFN-β promoter activity, and one of them is hnRNPA2B1. In mammalian cells, hnRNPA2B1 initiates and amplifies the innate immune response to DNA virus by translocating to the cytoplasm, where it then activates the TBK1-IRF3 pathway (34). Whether hnRNPA2B1 can regulate the RLR signaling pathway is unknown. Chicken hnRNPA2B1 possibly regulates the chIFN-β production by targeting chMDA5, but this hypothesis needs further study. We further investigated 10 proteins (i.e., DDX5, HSPA8, hnRNPH2, EEF1A1, EEF1A1, RBM39, HSP70, HSPA5, PABPC1, and PRDX1) that significantly regulated the chIFN-β promoter activity stimulated by chMDA5 overexpression and H5N6 virus infection and found that chicken DDX5, HSPA8, HSP70, hnRNPH2, IFIT5, and PRDX1 could interact with chMDA5 (Figure 4). DDX5 is a member of the DEAD box family of RNA helicases. Mammalian DDX46, one of the DEAD box family proteins, inhibits innate immunity by entrapping m^6^A-demethylated RNA of MAVS, TRAF3, and TRAF6 in the nucleus (35). In the present study, we observed that chicken DDX5 was mainly distributed in the cytoplasm, and the association between chicken DDX5 and chMDA5 occurred in the cytoplasm (Figure 4A). Mammalian DDX5 primarily functions in the nucleus (36, 37). It is unclear how DDX5 elucidates its function to regulate the chMDA5-mediated signaling pathway in the cytoplasm. Given that DDX5 could bind to the CARDs of chMDA5, this gene would probably disrupt the association between chMDA5 and MAVS. The role of chicken DDX5 in the chMDA5-mediated signaling pathway deserves further investigation. Another protein of interest is chicken HSPA8, which interacts with chMDA5-N. Mammalian HSPA8 interacts with MAVS and impairs the formation of MAVS aggregates to inhibit antiviral response (32). We did not examine whether chicken HSPA8 could interact with chicken MAVS. However, we propose that chicken HSPA8 could function as a negative regulator for the chMDA5-mediated signaling pathway by impairing the association between chMDA5 and MAVS; therefore, it is of great interest to investigate the influence of chicken HSPA8 on chicken MAVS aggregates upon virus infection.

Among proteins that interacted with chMDA5-N, we focused on chicken hnRNPH2, a member of the hnRNP family of proteins that complex with heterogeneous nuclear RNA. Evidence suggests that chicken hnRNPH2 inhibited the chIFN-β production, probably mainly through impairing chMDA5–MAVS association (Figure 6B). In particular, human hnRNPM inhibits RNA virus-triggered signaling by interacting with RIG-I and MDA5 and impairing the binding of the RLRs to viral RNA (26). It has been demonstrated that human hnRNPM underwent a shift from the nucleus to the cytoplasm upon RNA virus infection. In this study, we observed that chicken hnRNPH2 was distributed in both the cytoplasm and the nucleus. In the cytoplasm, co-location of chMDA5-N and chicken hnRNPH2 was found, which was in accordance with the function of chMDA5 in the cytoplasm. However, whether chicken hnRNPH2 could undergo nucleus–cytoplasm trafficking upon virus infection, or other stimulation such as IFN-β, needs further exploration.

## Data Availability Statement

All datasets generated for this study are included in the article/Supplementary Material.

## Author Contributions

XL conceived and designed the experiments. XL, SY, HM, and PR performed the experiments and analyzed the data. XL and MJ wrote the manuscript. All authors reviewed, revised, and approved the final manuscript.

## Conflict of Interest

The authors declare that the research was conducted in the absence of any commercial or financial relationships that could be construed as a potential conflict of interest.

## Footnotes

1 http://string-db.org
