# Supplementary material for: hnRNPH2 as an Inhibitor of Chicken MDA5-Mediated Type I Interferon Response: Analysis Using Chicken MDA5–Host Interactome
Source: Front Immunol. 2020 Oct 6;11:541267. doi: 10.3389/fimmu.2020.541267 (PMC7573076; doi:10.3389/fimmu.2020.541267)
Supplement: Supplementary file 1 [file Data_Sheet_1.PDF]

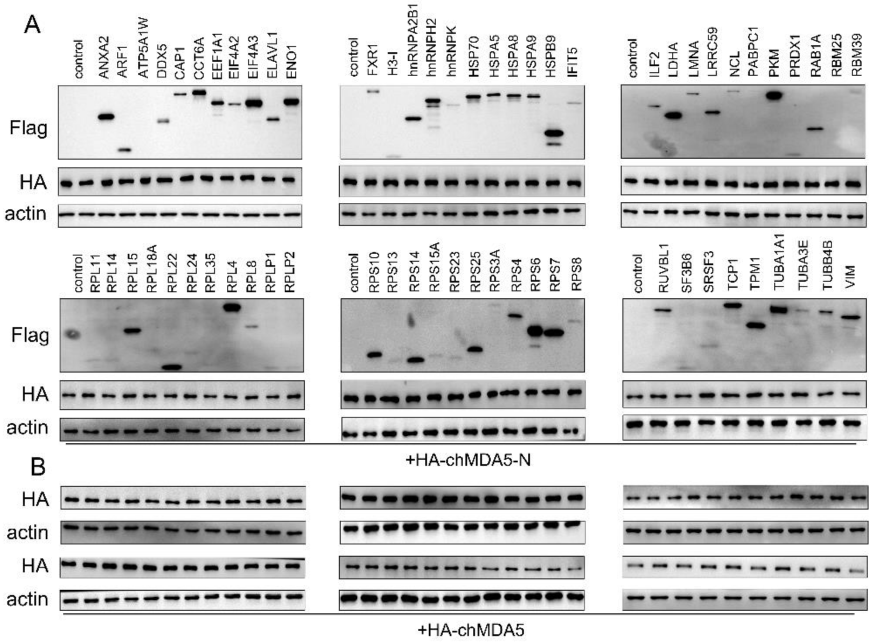

Figure S1. The expression of transfected genes

DF1 cells in 12-well plate were cotransfected with 0.5 μg of pCAGGS–chMDA5-N (A) or pCAGGS–chMDA5 (B) and 0.5 μg of genes that were cloned into expression vector pCMV14-3Flag, together with 0.2 μg of chIFN-luc and 10 ng internal control Renilla. Cells were lysed for firefly and Renilla luciferase activities test 24 h later. And the corresponding protein in the lysates were also detected by western blot. The sample order in (B) is as same in (A).

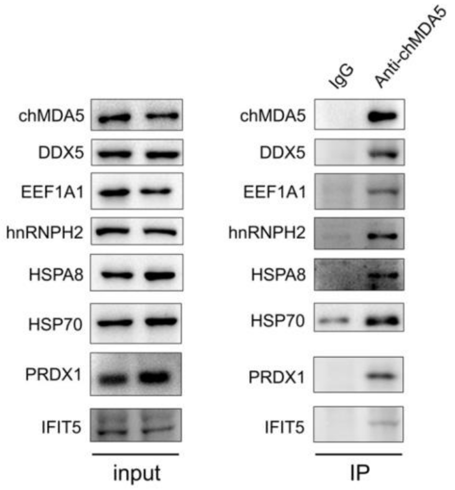

Figure S2. Endogenous interaction of chMDA5 and the selected six chicken proteins

DF1 cells in two 10-cm dish were lysed in RIPA buffer with EDTA-free protease inhibitor cocktail for 30 min at 4 °C. The cleared supernatants were incubated with anti-MDA5 antibodies or control IgG at 4 °C for 1 h, and then the antibody-antigen complexes were incubated with protein A/G at 4 °C overnight. After four times washes

with lysis buffer, the elution was subjected to western blot with corresponding antibodies.

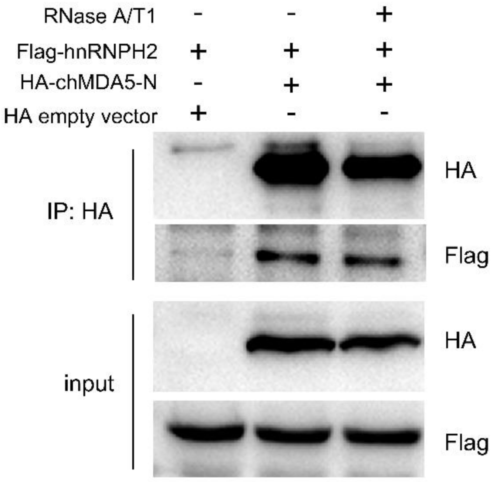

Figure S3. The interaction of chMDA5-N with hnRNPH2 is independent on RNA
1 µg of HA-tagged chMDA5-N and 1 µg of Flag-tagged indicated chicken proteins were co-transfected into DF1 cells grown in six-well plate. 24 h later, total proteins were extracted using RIPA buffer, and the cleared lysates were treated with 100 µl RNase A/T1 (EN0551, Thermo) at 37 °C for 20 min, followed by standard Co-IP assay and western blot, as described in Methods
